# Rehabilitation of Upper Extremity by Telerehabilitation Combined With Exergames in Survivors of Chronic Stroke: Preliminary Findings From a Feasibility Clinical Trial

**DOI:** 10.2196/33745

**Published:** 2022-06-22

**Authors:** Dorra Rakia Allegue, Johanne Higgins, Shane N Sweet, Philippe S Archambault, Francois Michaud, William Miller, Michel Tousignant, Dahlia Kairy

**Affiliations:** 1 School of Rehabilitation Université de Montréal Montreal, QC Canada; 2 The Centre for Interdisciplinary Research in Rehabilitation of Greater Montreal Institut universitaire sur la réadaptation en déficience physique de Montréal Montreal, QC Canada; 3 Mission Universitaire de Tunisie Montreal, QC Canada; 4 Department of Kinesiology and Physical Education McGill University Montreal, QC Canada; 5 School of Physical & Occupational Therapy McGill University Montreal, QC Canada; 6 Department of Electrical Engineering and Computer Engineering Université de Sherbrooke Sherbrooke, QC Canada; 7 Department of Occupational Science & Occupational Therapy University of British Columbia Vancouver, BC Canada; 8 Faculty of Medicine and Health Sciences, School of Rehabilitation Université de Sherbrooke Sherbrooke, QC Canada; 9 Center of research on Aging Sherbrooke, QC Canada

**Keywords:** stroke, rehabilitation, virtual reality, video games, telerehabilitation, upper extremity, motivation, mHealth, mobile health, personalized care, stroke rehabilitation

## Abstract

**Background:**

Exergames are increasingly being used among survivors of stroke with chronic upper extremity (UE) sequelae to continue exercising at home after discharge and maintain activity levels. The use of virtual reality exergames combined with a telerehabilitation app (VirTele) may be an interesting alternative to rehabilitate the UE sequelae in survivors of chronic stroke while allowing for ongoing monitoring with a clinician.

**Objective:**

This study aimed to determine the feasibility of using VirTele in survivors of chronic stroke at home and explore the impact of VirTele on UE motor function, quantity and quality of use, quality of life, and motivation in survivors of chronic stroke compared with conventional therapy.

**Methods:**

This study was a 2-arm feasibility clinical trial. Eligible participants were randomly allocated to an experimental group (receiving VirTele for 8 weeks) or a control group (receiving conventional therapy for 8 weeks). Feasibility was measured from the exergame and intervention logs completed by the clinician. Outcome measurements included the Fugl-Meyer Assessment-UE, Motor Activity Log-30, Stroke Impact Scale-16, and Treatment Self-Regulation Questionnaire-15, which were administered to both groups at four time points: time point 1 (T1; before starting the intervention), time point 2 (after the intervention), time point 3 (1 month after the intervention), and time point 4 (T4; 2 months after the intervention).

**Results:**

A total of 11 survivors of stroke were randomized and allocated to an experimental or a control group. At the onset of the COVID-19 pandemic, participants pursued the allocated treatment for 3 months instead of 8 weeks. VirTele intervention dose was captured in terms of time spent on exergames, frequency of use of exergames, total number of successful repetitions, and frequency of videoconference sessions. Technical issues included the loss of passwords, internet issues, updates of the system, and problems with the avatar. Overall, most survivors of stroke found the technology easy to use and useful, except for 9% (1/11) of participants. For the Fugl-Meyer Assessment-UE and Motor Activity Log-30, both groups exhibited an improvement in >50% of the participants, which was maintained over time (from time point 3 to T4). Regarding Stroke Impact Scale-16 scores, the control group reported improvement in activities of daily life (3/5, 60%), hand function (5/5, 100%), and mobility (2/5, 40%), whereas the experimental group reported varied and inconclusive results (from T1 to T4). For the Treatment Self-Regulation Questionnaire-15, 75% (3/4) of the experimental group demonstrated an increase in the autonomous motivation score (from T1 to time point 2), whereas, in the control group, this improvement was observed in only 9% (1/11) of participants.

**Conclusions:**

The VirTele intervention constitutes another therapeutic alternative, in addition to conventional therapy, to deliver an intense personalized rehabilitation program for survivors of chronic stroke with UE sequelae.

**International Registered Report Identifier (IRRID):**

RR2-10.2196/14629

## Introduction

### Background

Many survivors of stroke experience sequelae in the upper extremity (UE; eg, weakness, loss of coordination, and nonuse syndrome) [1], which may affect activities of daily living in the long term [2]. Exergames are increasingly being used among survivors of stroke for different functional skills (eg, physical activity, UE exercises, mobility, and balance) in various practice settings (eg, rehabilitation centers, hospitals, clinics, community health centers, and homes) [3]. Given the chronic nature of stroke, exergames present a relevant solution to continue exercising at home after discharge to maintain physical function and activity levels.

### Exergames: Types and Efficacy

Two main types of exergames have been described in the literature: commercially available off-the-shelf systems and customized systems [4,5]. Commercially available off-the-shelf systems, such as Nintendo Wii [6], Sony Playstation EyeToy games [7], Xbox 360 Kinect [8], and new technologies (the Xbox Series X [9] and Xbox one X [10]) present simple solutions for real-time video capture at a low cost (Xbox 360 costs US $250) [11], which encourages their adoption in clinical studies, especially when home interventions are considered [5].

Customized systems are generally designed through research and use cutting-edge technology to create a virtual environment, such as the Computer Assisted Rehabilitation Environment (Motek) [12] or the Interactive Rehabilitation Exercise (IREX; Gesture Tek) System 2D [13]. Compared with commercially available off-the-shelf systems, these technologies offer personalized game settings (speed, range of movement, and number of repetitions). Indeed, environments of customized exergames offer conditions of practice similar to those of the physical world, allowing task-specific activities (eg, in IREX, placing boxes on different shelves, catching a ball instead of a soccer goalkeeper, and juggling balls) and mass repetition of the movement, which may promote neuroplasticity [14].

Although customized exergames can be expensive (eg, the price of the IREX system can cost >US $15,000) and accessible only through specialized rehabilitation centers (eg, the Computer Assisted Rehabilitation Environment requires a large space and supervision) [15], some customized commercial systems can be more accessible to the population and only require a readily available Kinect camera to capture movement, in addition to computer and internet access [4], such as Doctor Kinetic (Doctor Kinetic), SaeboVR (Saebo), VirtualRehab (Evolv), and Jintronix (Jintronix).

A recent meta-analysis by Aminov et al [16] showed statistically significant efficacy of both types of exergames (customized vs commercially available off-the-shelf systems) in improving UE motor function (eg, Fugl-Meyer Score), activity (eg, Box and Blocks Test), and social participation (eg, Motor Activity Log-30 [MAL-30]) when compared with conventional therapy. Commercially available systems demonstrated a low mean effect size (Hedges g 0.33, 95% CI 0.14-0.51; *P*=.01), whereas customized exergames showed a moderate mean effect size (Hedges g 0.58, 95% CI 0.41-0.76; *P*=.01) [16]. During the follow-up periods (4-6 weeks and 8-26 weeks), the authors observed maintenance of these gains with weak to moderate effects on function and activity, and small to nonsignificant effects on social participation [16].

Overall, exergames offer several advantages compared with conventional therapy (eg, mass repetitions, feedback on activity, and motivation), which could explain the efficacy of these interventions in several metanalyses [15-20]. Several neuroscience studies have highlighted the ability of virtual reality (VR) to stimulate motor learning in the context of stroke [14,21,22]. Moreover, Maier et al [19] explained the superior efficacy of customized exergames compared with commercially available systems based on the presence of more elements promoting neuroplasticity (in 11/22, 50% of studies using customized systems), such as varied practice, feedback (eg, score, encouragement, and real-time visualization of the hand), increasing difficulty, or specific task practice. Given the promising potential of customized exergames, it is worthwhile to consider implementing them at the homes of survivors of stroke to optimize the recovery of persistent UE sequelae and maintain gains over time.

### Telerehabilitation Combined With Exergames

Telerehabilitation refers to the use of information and communication technology that provides remote rehabilitation [23]. Considering the context of the COVID-19 pandemic, telerehabilitation has been ideal to maintain the provision of rehabilitation services to those who need it most (older adults, people with difficulty accessing rehabilitation services, and people with deficits). The use of customized exergames combined with telerehabilitation may be an interesting alternative for rehabilitating UE deficits in survivors of chronic stroke while allowing for ongoing monitoring. When considering home interventions, exergames were usually provided with no supervision [24,25] or only follow-up sessions by telephone [26-28], which may have left the window open to compensation, mismatch of difficulty progression and improvement, a decrease in motivation [29], and feelings of loneliness [30]. In addition, exergames using the Kinect camera aimed at UE rehabilitation mainly offer exercises for the shoulder and elbow, with no emphasis on hand exercises. For example, the Kinect camera in the Jintronix exergame does not detect the hand and fingers; therefore, specific hand exercises are not provided [31]. Thus, the use of VR and customized exergames combined with telerehabilitation (eg, VirTele) is particularly relevant for providing a survivor of stroke–centered and exergame-based rehabilitation program [32,33]. The VirTele technology was previously tested with a survivor of stroke and was shown to be feasible for use in remote UE rehabilitation, which helped inform this study’s protocol [33]. The preliminary efficacy results showed improvement in UE motor function, quantity and quality of use, and impact on quality of life, along with a high level of autonomous motivation [33], hence the interest in continuing to study the VirTele intervention with more participants. In addition, given the novelty of VirTele, information on the optimal dose, time since stroke, and criteria for identifying participants who may benefit the most from VirTele is needed.

Therefore, it is necessary to conduct a feasibility clinical trial to (1) determine the feasibility of using VirTele with survivors of chronic stroke at home and (2) explore the impact of VirTele on UE motor function, quantity and quality of use, quality of life, and motivation in survivors of chronic stroke compared with conventional therapy.

## Methods

### Study Design

This study was a 2-arm feasibility clinical trial. Until the study could be pursued, considering the rapid progress of VR and telerehabilitation technologies, we considered it relevant to present the findings collected during the first 9 months (before the onset of the COVID-19 pandemic) to inform future technology development and implementation.

Eligible participants were randomly allocated to an experimental group (receiving VirTele for 8 weeks) or a control group (receiving conventional therapy for 8 weeks). Block randomization (block size of 6) was used, given the time and access to materials (3 computers were available at a time). There were 42 phone inquiries, during which 29 potential participants were excluded. A total of 13 potential participants were assessed for eligibility by in-person screening, and 11 were retained and randomly allocated to the control or experimental groups.

Outcome measurements were administered to both groups at four time points: before starting the intervention (time point 1 [T1]), after the end of the 2-month intervention (time point 2 [T2]), 1 month later (time point 3 [T3]), and 2 months later (time point 4 [T4]). Research team members who were blinded to group assignment and not involved in the interventions (VirTele or conventional therapy) were responsible for the randomization. During the period of the COVID-19 pandemic, evaluators could not be blinded to the group assignment as the participants in the experimental group were evaluated using the telerehabilitation system used in VirTele intervention.

### Ethics Approval

Before enrollment, all participants provided informed consent. This feasibility clinical trial was registered at ClinicalTrials.gov (NCT03759106) and was approved by the Research Ethics Board of the Center for Interdisciplinary Research in Rehabilitation of Greater Montreal (review number CRIR-1319-0218) [32]. This study was conducted according to the CONSORT (Consolidated Standards of Reporting Trials) guidelines [34].

### Participant Selection and Recruitment Strategy

Participants were recruited from the archives of rehabilitation centers (offline via a database of potential participants) and the community situated in Montreal (via the ClinicaTtrials.gov website; Quebec, Canada) [32]. Eligible participants included survivors of stroke (ischemic or hemorrhagic) with residual UE impairment (Chedoke-McMaster arm component, scores 2-6), who stopped receiving rehabilitation services and were able to use the exergame system (eg, move the exergame avatar with the affected UE) [32]. Participants were excluded if they had severe cognitive or communication impairment, uncontrolled medical conditions (eg, cardiac condition), balance deficits, visual impairment, and UE mobility deficits (restricted movements or inability to move the avatar).

Eligibility was assessed by a research assistant. The study therapists included physiotherapists working in the Montreal area with experience in stroke rehabilitation.

### Intervention Protocol

#### Experimental Group

The experimental group received the VirTele program. VirTele is an 8-week home rehabilitation program that includes Jintronix exergames [31] for UE rehabilitation and the Reacts app (Technologies innovatrices d’imagerie and Reacts) [35] to conduct videoconference sessions with clinicians. The experimental group received the VirTele equipment at home which included a computer, a Kinect camera, the Reacts app, the Jintronix software, and a USB internet key (if needed). Before starting the intervention, participants, including clinicians and survivors of chronic stroke, received a 1-hour training session to familiarize themselves with the Jintronix exergames and the Reacts app [32].

The Jintronix exergames included 5 games for UE training (*Space Race*, *Fish Frenzy*, *Pop Clap*, *Catch and Carry an apple*, and *Kitchen clean-up*) [31]. The clinician adjusted the difficulty parameters of each game remotely (eg, speed, duration, number of repetitions, and direction of the trajectory) according to the participant’s preference and functional abilities. An automated log system of the participant’s performance during exergames was available on the Jintronix portal (eg, active time spent on exergames, scores, number of tasks completed, and amount of trunk compensation), allowing the clinician to monitor the participant’s progression. The training protocol included five 30-minute sessions of Jintronix exergames per week for 8 weeks, targeting 20 hours of training overall.

The Reacts app [35], a videoconferencing platform, was used by the clinician to schedule videoconference meetings synchronized with sessions when the survivor of stroke was playing exergames to, for example, supervise the participant’s performance, correct their posture, grade the difficulty based on performance, and match games to the participant’s preferences and needs. Furthermore, the Reacts app was also used by the clinician to administer motivational interviewing [36].

Motivational interviewing is a person-centered approach used in behavioral interventions, which comprises behavior change techniques (BCTs) [37] and relational techniques [36]. Motivational interviewing has also been associated with the self-determination theory (SDT) [38]. The SDT is an approach that highlights the importance of autonomy and engaging individuals in their decision-making processes [39]. According to the SDT, clinicians can create a social environment that fosters autonomy (volition in one’s actions), competence (belief in one’s actions), and relatedness (a sense of belonging), which are 3 dimensions that are essential for promoting autonomous motivation and well-being [39]. In line with the SDT, survivors of stroke were given greater autonomy in determining their program by being able to choose from a range of exercises, being involved in grading the difficulty level of the games, and identifying strategies to increase the use of their affected UE in the long term through self-directed exercises and daily activities (eg, using the affected UE for dressing). In addition to exergames, supplementary exercises targeting hand fine motor skills and UE were suggested by the clinician to meet the individual goals of the survivors of stroke.

The videoconferencing sessions were scheduled as follows: 3 times a week for the first 2 weeks, twice a week for the following 2 weeks, and then once a week for the remaining 4 weeks to maintain motivation, ensure that the exercises are adequately tailored, and identify strategies to maintain the activity level of the UE after the study ended.

The training of the VirTele group was conducted at the participant’s home after the installation of the equipment and lasted approximately 30 minutes to 1 hour. The training included a practical workshop on the use of the exergames and videoconferencing system. At the end of the training, a VirTele user manual (developed by the research team) was provided to the participants. Clinicians were trained in motivational interviewing [36] before the start of the study. A motivational interviewing guide (discussion plan) based on BCTs [37] and motivational techniques [36] was conceived by the research team and provided to the clinicians as a support tool that can help them choose strategies adapted to the client’s needs.

For further information regarding the Reacts app, Jintronix exergames, and motivational interviewing, refer to the published study protocol [32] or previous case studies exploring VirTele use among survivors of stroke [33,40].

#### Control Group

In Canada, survivors of chronic stroke receive the Graded Repetitive Arm Supplementary Program (GRASP) [41] as a home rehabilitation training program to exercise the affected UE and use it in activities of daily living [2]. Therefore, the control group received the GRASP, which included exercises for the arm and hand (strengthening and range of motion) and functional activities targeting the UE [41]. The GRASP equipment included various sizes of Lego and wooden blocks, poker chips, clothes pegs, popsicle sticks and toothpicks, paper clips of various sizes, various jars, a weight of 0.45 kg, tennis ball, foam ball, plastic cup, modeling clay, knife and fork, and a target board [41]. The control group was invited to perform the GRASP exercises for 8 weeks, 5 days per week (30-minute sessions), targeting 20 hours of exercise overall (same as the experimental group) [32]. The time spent on the GRASP program, the number of sessions, and events such as fatigue and pain were reported at T2 after the intervention was terminated. No follow-up was provided during the 8-week intervention period, similar to conventional therapy. However, at the end of the study, the participants were offered one session with the clinician to discuss strategies for improving the use of UE in activities of daily living [32]. All participants received a 30-minute training to familiarize themselves with the GRASP equipment and exercises [32].

### Outcomes Measures

#### Overview

At the start of the study, participant evaluations were conducted at the Center for Interdisciplinary Research in Rehabilitation of Greater Montreal in the presence of an evaluator. At the start of the COVID-19 pandemic, all research activities at the research site were suspended, and all evaluations were conducted remotely. For the experimental group, the evaluations were conducted using the Reacts videoconferencing system. For the control group, the evaluations were conducted either by phone or by a videoconferencing system available at the participant’s home.

#### Feasibility Indicators

Given the novelty of VirTele, the feasibility data collected for the experimental group included the number and active time spent on exergame sessions, frequency and time spent by the clinician during videoconferencing sessions, exercise adherence, and resource use (equipment and technical support). These were obtained directly from the Jintronix and Reacts systems, as well as from intervention logs completed by the clinician at the end of each session. Safety indicators, such as the occurrence of adverse events (eg, pain, fatigue, and dizziness), were documented by the clinician and technical team [32]. Information about technical difficulties was obtained from a log completed by the clinicians and technical team [32]. Satisfaction with the technology and the interaction between the clinician and the survivor of stroke were assessed using the Modified Short Feedback Questionnaire (adapted from Davis [42]) and the Health Care Climate Questionnaire (Perceived Autonomy Support) [43]. The Modified Short Feedback Questionnaire includes 2 subscales with 6 items, including a 7-point Likert-type scale ranging from *extremely likely* (score 1) to *extremely unlikely* (score 7). The first subscale evaluates the perceived usefulness (total score range 6-42; a lower score indicates that the technology is extremely useful) and the second subscale evaluates the perceived ease of use (total score range 6-42; a lower score indicates that the technology is extremely easy to use). The Health Care Climate Questionnaire includes 6 items with a 7-point Likert-type scale ranging from *strongly agree* (score 1) to *strongly disagree* (score 7), evaluating the need for support from the clinician, as perceived by the survivor of stroke (total score range 6-42; a high score indicates a higher perceived need for support from the clinician).

The process indicators were also documented to inform the validity of the study protocol and included data on recruitment rate (rate of participants per month and duration of recruitment) and retention rate (percentage of participants who completed the VirTele program) [32].

#### Performance Outcome Measure

The Fugl-Meyer Assessment-UE (FMA-UE) [44,45] motor function score was used as the primary outcome to evaluate UE motor function impairment. The FMA-UE motor function score [44,45] captures synergy, coordination, and sensorimotor functions (UE, wrist, and hand). The FMA-UE score has been shown to be valid in participants with stroke [46] and reliable for administration at a distance (video observation of an evaluator administering the FMA-UE on site) [47]. Given the COVID-19 pandemic, we tested the feasibility of administering the FMA-UE motor function at a distance with no on-site evaluator (video observation of the participant’s performance), which was pretested in a previous study with a first survivor of stroke [33]. With respect to the various collection methods (by videoconferencing, by telephone, or on site), the FMA-UE motor function score was adjusted to 60 in all participants (experimental and control groups) by eliminating the parts of the scale that could not be evaluated remotely (reflex activity component).

#### Self-reported Questionnaires

The secondary outcomes included the Motor Activity Log-30 (MAL-30) [48,49], the Stroke Impact Scale-16 (SIS-16) [50,51], and the Treatment Self-Regulation Questionnaire-15 (TSRQ-15) [52].

The MAL-30 captures both quality (MAL-30 quality of use) and quantity of use (MAL-30 amount of use) of the affected UE in 30 daily activities (eg, writing on paper, brushing teeth, and using a fork or spoon for eating) [48,49]. The MAL-30 is reliable and valid for the poststroke population [53].

The SIS-16 is a 16-item questionnaire that captures the impact of stroke on the quality of life regarding hand function, activities of daily living, and mobility [50,51]. The SIS-16 has demonstrated good reliability and validity [54].

The TSRQ-15, a 15-item questionnaire, captures different processes of motivation consistent with the SDT, including autonomous motivation, “where a person accepts changes and behaves autonomously”; amotivation or the “lack of motivation”; external regulation, “where a person behaves to obtain a reward, or avoid punishment”; and introjected regulation, “where a person behaves for pride or to avoid feeling guilty” [52]. The TSRQ-15 has demonstrated good reliability and validity across health care and rehabilitation contexts [52,55].

### Data Analysis

Descriptive statistics (means, frequencies, and SDs) were used to (1) describe the sociodemographic characteristics of survivors of chronic stroke in both groups (age, sex, dominance, time since stroke, type of stroke, side of stroke, Chedoke-McMaster UE score, living arrangement, and ability to use a computer), (2) report feasibility indicators (eg, time spent on exergames, frequency of use, total number of repetitions, number of videoconferencing sessions, satisfaction with the technology, and perceived autonomy support), and (3) report impact indicators (frequency of participants who improved and worsened for each outcome measure). All outcome measure changes were compared with their minimal clinically important differences (MCIDs) when applicable [32].

## Results

### Overview

**Figure 1:**
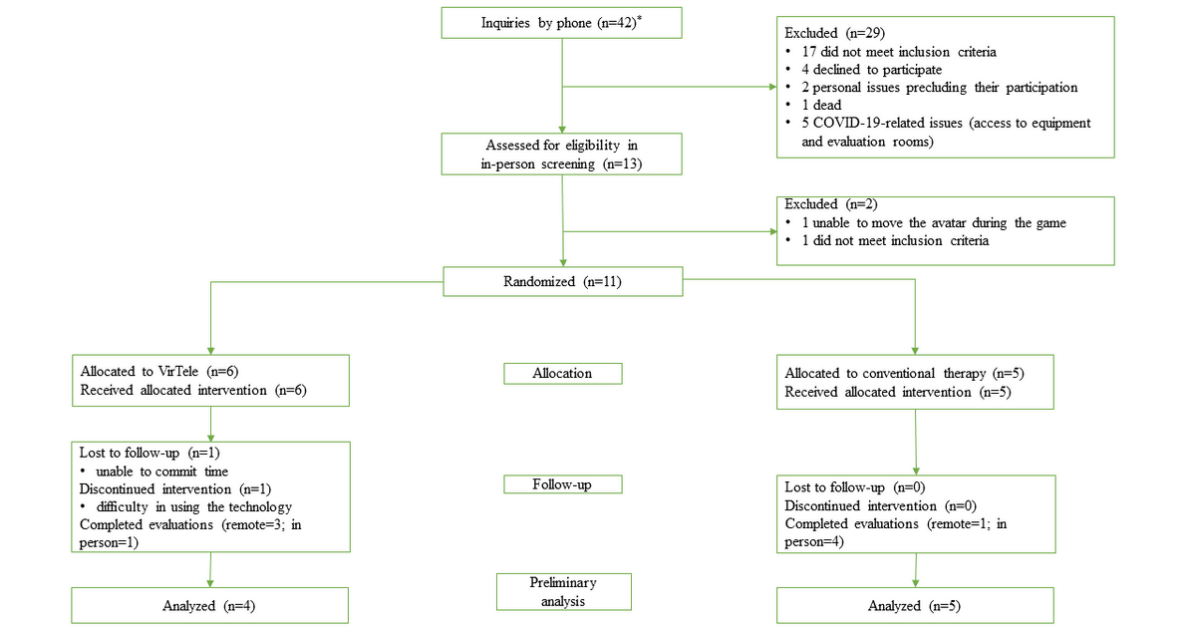
Group allocation, follow-up, and data analysis. *Recruitment was interrupted because of the COVID-19 pandemic.

As research activities were suspended because of the COVID-19 pandemic, data collection, as scheduled in the research protocol [32], was delayed and extended. A total of 11 survivors of stroke were randomized and allocated to a treatment group (VirTele intervention or conventional therapy). The attrition rate was 18% (2/11), as 2 participants from the VirTele group did not complete the study (Figure 1). One of the patients was lost at follow-up because of an inability to commit time, and one discontinued the VirTele intervention because of difficulties using technology (unable to use the mouse or the keyboard and to start the computer).

Approximately 50% (2/4) of participants in the experimental group and 20% (1/5) of participants in the control group received their allocated treatment at the onset of the COVID-19 pandemic in Canada (March 2021). At that time, every research activity was suspended, and outcome measurements at T2 could not be administered. Thus, participants were offered the opportunity to pursue the allocated treatment for 3 months instead of 8 weeks and were evaluated remotely at the end of the 3-month intervention (T2), a month later (T3), and 2 months later (T4). The sociodemographic data are provided in Table 1.

**Table 1:** Sociodemographic data (N=9).

Characteristics		VirTele group (n=4)	Control group (n=5)
Age (years), mean (SD)		57.8 (21.8)	56.4 (17.3)
**Sex, n (%)**			
	Male	2 (50)	2 (40)
	Female	2 (50)	3 (60)
**Hand dominance, n (%)**			
	Right	3 (75)	3 (60)
	Left	—^a^	2 (40)
	Ambidextrous	1 (25)	—
Time since stroke (years), mean (SD)		8 (2)	9.8 (3.0)
**Type of stroke, n (%)**			
	Hemorrhagic	1 (25)^b^	2 (40)
	Ischemic	2 (50)^b^	3 (60)
**Side of stroke, n (%)**			
	Right	4 (100)	3 (60)
	Left	0 (0)	2 (40)
Chedoke-McMaster UE^c^ score, mean (SD)		3.8 (1.0)	4.8 (1.3)
**Living arrangement, n (%)**			
	Living with family	3 (75)	2 (40)
	Living alone	1 (25)	3 (60)
**Ability to use a computer, n (%)**			
	Excellent	1 (25)	2 (40)
	Good	2 (50)	2 (40)
	Poor	1 (25)	1 (20)

^a^Not available.

^b^Information regarding the type of stroke was not available for participant ID11 at the time.

^c^UE: upper extremity.

### Feasibility Indicators

#### Process Indicators

Of the 42 inquiries by phone, 11 (26%) participants met the eligibility criteria and accepted to participate in the study. The rate of participant recruitment per month ranged from 0 to 6. In the VirTele group, 85% (5/6) of the participants completed the 8-week intervention (or the 3-month intervention during the COVID-19 pandemic). One of the participants discontinued the intervention because of persistent technical difficulties in accessing the VR system despite training (Figure 1).

#### Resources

The active time spent on exergames, the number of exergame sessions, the total number of repetitions, and activities performed in parallel to VirTele (which implies the use of UEs) of each participant receiving VirTele intervention are reported in Table 2.

The frequency of videoconference sessions varied between 9 and 11 sessions during the first 4 weeks (mean 9.8, SD 1.0), followed by 3 to 7 sessions during the second month (mean 5. SD 1.826) and 4 to 6 sessions during the third month (mean 5.0, SD 1.4).

The frequency of use and time spent on GRASP, as well as activities performed in parallel with GRASP (which implied the use of UEs), are reported in Table 3. Participant ID4, who did not use the GRASP during the 8-week intervention, reported that he was discouraged by the program as it mainly focused on his hand and wrist, which he could not move anymore since the stroke. Although participant ID9 received GRASP for 3 months, he only used the program for 6 weeks.

**Table 2:** Exergame sessions and activities performed by each participant receiving VirTele intervention.

Participant ID and total	Time spent on exergames (hours)			Frequency of use of exergames		Total number of repetitions^a^			Activities (other than those provided in VirTele)
	2 months	Third month			2 months		Third month		
ID1	18	N/A^b^	84		17,101		N/A	Stretching, cooking, and housework (cleaning and laundry)	
ID5	20	N/A	49		12,854		N/A	Exercises	
ID10	15.03	14.28	59		13,130		18,759	Housework (cleaning and laundry)	
ID11	13.18	4.23	58		11,649		5312	Housework (cleaning and laundry)	
Total, mean (SD)	16.6 (3.0)	9.2 (7.1)	62.5 (15.0)		13,683 (2367)		12,035 (9508)	—^c^	

^a^Reflects the number of successful tasks or movements completed during the exergame.

^b^N/A: not applicable.

^c^Not available.

**Table 3:** Frequency of use and time spent on GRASP^a^ in the control group.

Participant ID and total	Time spent on GRASP (hours)			Frequency of use of GRASP		Activities (other than GRASP)
	2 months	Third month				
ID3	4	N/A^b^	16		Bodybuilding, housework, and cooking	
ID4	0	N/A	0		Tennis (nonaffected hand), cooking, and housework	
ID6	8	N/A	16		Swimming, exercises, and housework	
ID7	84	N/A	56		Housework, using a computer, and exercises	
ID9	30	0	30		Gardening and shopping	
Total, mean (SD)	25.2 (34.86)	0 (0)	23.6 (20.99)		—^c^	

^a^GRASP: Graded Repetitive Arm Supplementary Program.

^b^N/A: not applicable.

^c^Not available.

#### Management

Technical issues were reported from the clinicians’ logs and included loss of password (to access the Reacts app) by the participant, internet issues, update of the system, sound or video cut off, and problems with the avatar (did not follow the movements of the UE). Technical issues were mainly managed by the clinician, the participant, or the participant’s caregiver. The technical team intervened once on site to deliver a 3G key, as the participant had no more internet access, and once by telephone with a participant to help them recover their passwords.

The clinicians’ logs showed that BCTs and motivational techniques were applied during the VirTele intervention with each participant in the experimental group. Among the 4 participants who completed the VirTele intervention, 3 (75%) participants (ID1, ID10, and ID11) reported more frequent use of the affected UE in activities of daily life and self-directed exercises (during the intervention), and 3 (75%) participants (ID1, ID5, and ID10) maintained the use of the affected UE after the intervention was terminated.

#### Scientific Feasibility

The 4 participants in the experimental group reported fatigue of the affected UE, which was managed by the clinician (by suggesting rest and stretching postures). Participant ID10 reported an increase in pain in the less-affected UE during the third month of VirTele; however, it did not seem to affect his adherence to the intervention, as recorded in the automatic logs accessible in the Jintronix portal (executed 59 sessions of exergames and spent 14 hours playing during the third month; Table 2).

The Health Care Climate Questionnaire showed a high score for perceived autonomy support in the experimental group (mean score 41.0, SD 1.7). Regarding the results of the perceived usefulness and perceived ease of use, most participants (3/4, 75%) found the technology extremely easy to use (mean score 11.0, SD 6.6) and extremely or quite useful (mean score 13.8, SD 15.5). Participant ID5 found the technology extremely or quite difficult to use (score 37/42) and slightly useful (score 20/42).

### Performance Outcome Measure

For the primary outcome (FMA-UE motor function score), 50% (2/4 in each group) exhibited an improvement with important change scores equal to or within the MCID ranges (between 4.25 and 7.25), maintained over time from 1 (T3) to 2 months (T4) after the intervention (Table 4). Participant ID9 in the control group could not be evaluated as the FMA-UE could not be administered by phone (the only technology used by the participant) during the COVID-19 pandemic.

**Table 4:** Fugl-Meyer Assessment–Upper Extremity motor function score in the experimental and control groups.

Group and participant ID		Fugl-Meyer Assessment–Upper Extremity motor function score (0-60)			
		Time point 1	Time point 2	Time point 3	Time point 4
**Experimental group**					
	ID1	24	31	28	41
	ID5	50	43	48	51
	ID10	18	14	18	18
	ID 11	25	29	31	24
**Control group**					
	ID3	43	48	42	41
	ID4	4	9	8	6
	ID6	46	47	57	59
	ID7	52	42	46	46

### Self-reported Questionnaires

#### MAL-30 Questionnaire

Regarding the MAL-30 quantity of use, 100 % (4/4) of all participants in the experimental group exhibited improvement from baseline (T1) to postintervention (T2), with the maintenance of benefits over time from 1 (T3) to 2 months (T4) after the intervention, whereas the control group showed improvement in 80% (4/5) of the participants from baseline (T1) to postintervention (T2), with maintained gains over time from 1 (T3) to 2 months (T4) after the intervention (Table 5). The MCID of the MAL-30 quantity of use was not available at that time.

For the MAL-30 quality of use, all participants in the experimental (4/4, 100%) and the control (5/5, 100%) groups demonstrated improvement from baseline (T1) to postintervention (T2), maintained over time from 1 (T3) to 2 months (T4) after the intervention, 2 of which reached the MCID (between 1.0 and 1.1; Table 5) [49].

**Table 5:** Motor Activity Log-30 scores in the experimental and control groups.

Group and participant ID		Motor Activity Log-30: quantity and quality of use of the affected upper extremity								
		Score quantity of use (from 0 to 5)					Score quality of use (from 0 to 5)			
		Time point 1	Time point 2	Time point 3	Time point 4	Time point 1		Time point 2	Time point 3	Time point 4
**Experimental group**										
	ID1	0.26	0.87	0.63	0.98	0.26		0.93	0.70	0.78
	ID5	1.64	2.07	2.91	2.71	1.32		1.68	2.45	2.34
	ID10	0.10	1.13	0.63	0.53	0.13		1.17	0.90	0.47
	ID 11	0.00	0.34	0.36	0.32	0.00		0.38	0.41	0.38
**Control group**										
	ID3	0.70	0.57	1.00	1.13	0.83		1.30	1.06	1.18
	ID4	0.00	0.07	0.07	0.10	0.00		0.03	0.07	0.10
	ID6	1.86	3.19	3.14	3.36	2.10		3.34	3.39	3.86
	ID7	1.21	2.69	2.64	3.14	1.52		2.72	2.58	2.93
	ID9	0.03	0.20	0.22	0.22	0.01		0.31	0.38	0.24

#### SIS-16 Questionnaire

For the SIS-16 hand function, only one of the participants in the experimental group exhibited improvement for the item “carry heavy objects (eg, bag of groceries),” with a score higher than the MCID (between 9.4 and 14.1) [51]. All participants in the control group (5/5, 100%) demonstrated improvement from baseline (T1) to postintervention (T2), maintained over time from 1 (T3) to 2 months (T4) after the intervention, with all scores higher than the MCID (Table 6).

**Table 6:** SIS-16^a^ scores in the experimental and control groups.

Group and participant ID		SIS-16 hand function (from 0 to 100)					SIS-16 activities of daily life (from 0 to 100)					SIS-16 mobility (from 0 to 100)			
		T1^b^	T2^c^	T3^d^	T4^e^	T1		T2	T3	T4	T1		T2	T3	T4
**Experimental group**															
	ID1	100	100	100	100	100		100	100	100	96		100	100	100
	ID5	0	0	25	0	47		50	53	38	46		61	46	54
	ID10	75	75	0	0	97		81	88	78	82		82	57	61
	ID 11	75	50	50	50	69		72	78	75	75		68	79	79
**Control group**															
	ID3	25	25	25	50	84		88	91	94	82		100	89	93
	ID4	75	75	100	75	91		75	81	81	89		86	96	93
	ID6	0	25	50	50	56		63	78	69	61		75	71	75
	ID7	25	50	100	100	100		97	97	94	100		100	96	100
	ID9	0	50	50	50	66		63	84	94	68		50	75	68

^a^SIS-16: Stroke Impact Scale-16.

^b^T1: time point 1.

^c^T2: time point 2.

^d^T3: time point 3.

^e^T4: time point 4.

For the SIS-16 activities of daily life, 50% (2/4) of the participants in the experimental group demonstrated improvement from baseline (T1) to postintervention (T2), maintained over time from 1 (T3) to 2 months (T4) after the intervention in only 1 participant (MCID was not detected). In the control group, 60% (3/5) of the participants exhibited improvement higher or within the MCID from baseline (T1) to 2 months after the intervention (T4; Table 6).

Regarding the SIS-16 mobility, 50% (2/4) of participants in the experimental group exhibited improvement from baseline (T1) to postintervention (T2), maintained over time from 1 (T3) to 2 months (T4) after the intervention, with a score higher than the MCID in only 1 participant. In the control group, 40% (2/5) of the participants exhibited improvement from baseline (T1) to postintervention (T2), maintained over time from 1 (T3) to 2 months (T4) after the intervention, with scores within or higher than the MCID (Table 6).

#### TSRQ Measure

In the experimental group, 75% (3/4) of the participants demonstrated an increase in their autonomous motivation score from baseline (T1) to 2 months after the intervention (T4). Further examination of the regulations that define the controlled motivation in the experimental group showed an increase in introjected regulation from baseline (T1) to postintervention (T2) in 75% (3/4) of the participants, maintained over time from 1 (T3) to 2 months (T4) after the intervention in only 1 participant. In parallel, the external regulation showed an increase of 75% (3/4) in the participants from baseline (T1) to postintervention (T2), with a tendency to decrease in the follow-up period from 1 (T3) to 2 months (T4) after the intervention. The motivation score was substantially low in all participants at all times (Table 7).

In the control group, only one of the participants demonstrated an increase in autonomous motivation from baseline (T1) to postintervention (T2), maintained over time from 1 (T3) to 2 months (T4) after the intervention. The examination of the introjected regulation showed substantially no change from baseline (T1) to postintervention (T2) in 80% (4/5) of the participants. The external regulation scores showed a tendency of increase in 40% (2/5) of the participants, maintained over time from 1 (T3) to 2 months (T4) after the intervention in only 1 participant. One of the participants showed a decrease from baseline (T1) to 2 months after the intervention (T4) in both introjected and external regulations. Amotivation scores tended to increase in 80% (4/5) of participants (Table 7).

**Table 7:** Treatment self-regulation scores in the experimental and control groups.

Group and participant ID		Autonomous motivation					Introjected regulation					External regulation					Amotivation			
		T1^a^	T2^b^	T3^c^	T4^d^	T1		T2	T3	T4	T1		T2	T3	T4	T1		T2	T3	T4
**Experimental g** **roup**																				
	ID1	42	42	42	43	14		14	14	8	4		10	4	4	3		3	3	3
	ID5	34	41	41	36	10		14	11	8	15		19	20	4	9		9	8	9
	ID10	42	42	42	42	2		8	14	12	16		11	16	22	9		6	9	9
	ID 11	19	27	24	24	5		10	5	6	9		13	12	11	8		7	4	11
**Control g** **roup**																				
	ID3	38	40	42	40	2		2	3	2	4		7	7	7	3		7	9	3
	ID4	40	39	42	39	10		11	11	11	8		6	17	12	6		6	8	8
	ID6	40	27	40	39	10		2	2	2	11		4	5	7	6		9	3	9
	ID7	40	32	33	33	13		13	13	2	24		10	16	4	9		3	8	3
	ID9	30	24	21	24	9		8	2	2	6		10	4	4	6		3	9	9

^a^T1: time point 1.

^b^T2: time point 2.

^c^T3: time point 3.

^d^T4: time point 4.

## Discussion

The objectives of this feasibility clinical trial were to (1) determine the feasibility of using VirTele with survivors of chronic stroke at home and (2) explore the impact of VirTele on UE motor function, quantity, quality of use, quality of life, and motivation in survivors of chronic stroke compared with conventional therapy.

### Feasibility and Impact Indicators

#### Feasibility Indicators

##### Criteria of VirTele Use

The results of this study suggest that VirTele is feasible to use at home among survivors of chronic stroke, aged 41 to 89 (mean 56.8, SD 21.8) years with 8 (SD 2) years since the stroke. However, certain criteria should be respected to benefit as much as possible from this technology, such as minimum knowledge of using computers (how to use a mouse and keyboard) or having a caregiver who is comfortable with computers and no severe aphasia that limits communication between the clinician and the survivor of the stroke. Overall, most survivors of stroke found the technology easy to use and useful, except for one of the participants.

The training provided before starting the intervention seems adequate for survivors of chronic stroke who have used a computer before but should be adjusted to better prepare participants who are not familiar with computers (never used before), such as a longer period of familiarization and personalized training. Although the clinicians reported no difficulties regarding technology use, novice clinicians may require support to address interoperability issues and acquire new skills (eg, choose exergames based on client capacities and goals, create exergame-based rehabilitation programs, select appropriate clients, and grade difficulty levels) to enhance their self-efficacy during practice [3].

##### Dose of the VirTele Intervention

In the context of this study, VirTele intervention dose was captured in terms of time spent on exergames (2 months: mean 16.6, SD 3.0 hours; third month: mean 9.3, SD 7.1 hours), frequency of use (mean 62,5, range 49-84 sessions), and the total number of successful repetitions (2 months: mean 13,683, SD 2367; third month: mean 12,035.5, SD 9508.46). Interestingly, dose in terms of time spent on exergames and frequency of use did not seem to have any moderating effect on FMA-UE and SIS-16 scores, which echo the findings of a previous study that found no advantages for higher dosing (duration and frequency of use) of VR approaches on rehabilitation outcomes (eg, FMA-UE, box, block) [16]. However, the performance of approximately 17,000 repetitions of successful tasks or movements during exergames appears to be the gold standard for achieving clinical improvements in UE motor function. Although participant ID10 attained 30,000 repetitions, no improvement was observed in the FMA-UE, which suggests that intense repetition is not always the gold key to recovery, as reported in a previous study (where UE improvement was attained following 30,341 repetitions) [33]. Furthermore, participant ID10 reported increased fatigue in the affected UE and pain in the less-affected UE, which reflects symptoms of overexercising and may prevent or reduce potential improvement. An evaluation of the FMA-UE score after the 8-week intervention in participant ID10 could have provided a better indicator of the UE motor condition (before the onset of symptoms at the third month).

Although all participants in the experimental group improved their MAL-30 scores, the MCIDs were only detected in participants ID5 and ID10, who spent the longest time on exergames (range 20-29 hours), which suggests a potential link between doses in terms of time spent on exergames and clinical improvement at the MAL-30. Previous studies conducted by Levin et al [56] (delivered 6.8 hours of video capture exergames) and Housman et al [57] (delivered 24 hours of gravity-supported exergames) intending UE rehabilitation in survivors of chronic stroke found no change and significant improvement, respectively, sustained at 6 months on the MAL scores. These findings suggest that longer exposure to exergames may lead to better outcomes in participation in real-life activities and support the potential transfer of gains from the virtual environment to physical real-life activities.

##### The Optimal Duration of the VirTele Intervention

The optimal effective duration of VirTele intervention (8 or 12 weeks) is not yet clear, considering the varied results of the primary and secondary outcomes between participants in the experimental group. However, it is worth noting that the total number of repetitions and frequency of use of the technology are not always affected by VirTele duration. For example, participant ID1, who used VirTele for 8 weeks, achieved a higher dose of repetition and frequency of use of the exergames than participant ID11, who used VirTele for 3 months. Further examination of the level of amotivation at baseline showed that participants with the lowest level of amotivation (ie, high motivation) had the highest dose of repetition and frequency of use during the first 8 weeks. This may suggest that motivation should be evaluated before starting the VirTele intervention to determine the adequate duration (8 or 12 weeks) necessary to achieve a high dose of repetition and frequency of use and that an appropriate motivational strategy should be provided to individuals who are amotivated.

##### Factors That May Affect Adherence to VirTele Intervention

During the first 8-week intervention period, female participants (ID1 and ID5) achieved the highest level of adherence to exergames compared with male participants (ID10 and ID11), which suggests that sex may play a role in choosing to play or not the VirTele exergames. A previous study [58] conducted on healthy participants aged 18 to 51 (mean 21.65, SD 4.43) years, showed that women preferred physically internet-based games compared with men, which may explain the higher level of adherence to VirTele exergames in women, although it should be carefully interpreted, considering the small sample size and other factors related to motivation and stroke (eg, UE weakness and pain), which may affect adherence to the system.

Age did not seem to affect adherence to the VirTele program, although lack of knowledge in information technology was often associated with older participants. Participant ID5, who was not familiar with information technology, had a caregiver who helped her use the system and was compliant with the VirTele program. However, previous experience in information technology may facilitate the use of this technology.

#### VirTele Impact Indicators

Regarding the primary outcome (FMA-UE), the experimental group reached the MCID from baseline (T1) to 2 months after the intervention (T4). This result is particularly relevant as the MCID was detected even if the total score of the scale was adjusted to 60, which supports the feasibility of administering the FMA-UE motor function remotely (without an evaluator on site). This result also supports the findings of a previous study [47] that examined the measurement properties of FMA-UE when administered remotely.

Regarding the secondary outcomes, both groups demonstrated improvement in the MAL-30 quantity and quality of use, which may suggest that the VirTele intervention is comparable with conventional therapy in terms of somatosensory information feedback, affecting the UE quality of movement. The supplementary exercises provided in VirTele (in addition to exergames) may have played a role in the integration of somatosensory information by manipulating real-life objects with force and tactile feedback, which are important for motor learning [14].

Regarding the quality of life (SIS-16 scores), the control group reported improvement in activities of daily life and hand function in 60% (3/5) and 100% (5/5) of the participants, respectively. In contrast, the experimental group reported varied and inconclusive results in terms of activities of daily life and hand function, despite the increased use of the UE (MAL-30 quantity) and improvement in the quality of use (MAL-30 quality). Participant ID1 reported a score of 100% (from T1 to T4) in SIS-16 hand function and activities of daily life, which indicates that no further gains can be achieved. Participant ID10 reported the appearance of pain in the less-affected UE (during the third month of VirTele intervention), which may have affected his performance during activities of daily life and the score of the SIS-16 hand function for the item “carry heavy objects (eg, bag of groceries)” as survivors of stroke often use compensatory strategies by the less-affected UE to help or assist the performance of the affected UE [59].

Further explanation of the difference between the two groups regarding the SIS-16 scores may be associated with the training paradigm; the GRASP mainly targeted the hand and wrist, with little focus on gross motor skills, whereas the VirTele intervention mainly targeted gross motor skills, with supplementary exercises for the hand. Thus, training with the GRASP might better meet individual needs when it comes to performance in activities that require fine motor skills, although both groups demonstrated improvements in the quality and quantity of use of the UE. This also suggests that combining VirTele with conventional therapy such as the GRASP may maximize the recovery potential, which echoes the findings of Laver et al [60] who determined that the use of VR combined with conventional therapy had a significant effect on UE outcomes compared with when it was used alone (not significant).

### Role of Motivational Interviewing

In the experimental group, 75% (3/4) of the participants demonstrated an increase in autonomous motivation compared with 20% (1/5) in the control group. In parallel, the experimental group demonstrated no change in the amotivation score, whereas the control group tended to show an increase in 80% (4/5) of participants. These results may suggest that VirTele intervention is more motivating than conventional therapy and that motivational interviewing delivered in the experimental group could have played a role in the development of autonomous motivation, which is important to maintain behavior changes of the UE.

Other factors that may stimulate autonomous motivation include enjoyment and improving skills [61]. In this context, VirTele exergames offer playful and varied exercises with different levels of difficulty that could give survivors of stroke a real feeling of competence and more confidence in their abilities when they manage to succeed. Furthermore, some components of exergames, such as visual and auditory feedback (encouragement, score of the game, and indication of successful vs unsuccessful movement) [62] and quality of graphics [30], may enhance the enjoyment of participants and increase autonomous motivation, which may affect adherence to exercise.

Furthermore, a multiple case study conducted with participants ID5, ID1, and ID11 showed that VirTele clinicians used many motivational interviewing strategies (BCTs and motivational techniques) that would support participants’ psychological needs [33]. Such an environment may lead to effective behavior changes [63], such as that experienced by participants ID5 and ID10 (high adherence to exergames and maintained use of the affected UE at the end of the VirTele intervention) [33]. In addition, the experimental group performed an enormous amount of repetition and had a higher frequency of use of the allocated treatment than the control group.

In contrast, participant ID11 did not express any intention to continue using the affected UE when the intervention was terminated, which may be explained by the miscommunication encountered between the participant and the respective clinician because of aphasia [33]. An interview with ID11’s clinician in the multiple case study showed that the latter had difficulty understanding the needs of the participant to provide adequate motivational support [33]. In addition, participant ID11 was ambidextrous, which may have increased the use of compensatory strategies by the less-affected UE.

### Limitations and Recommendations

The findings of this feasibility clinical trial should be carefully interpreted as some limitations were identified. First, the VirTele and GRASP interventions presented different training paradigms (gross and fine motor skills); however, only gross motor skills (coordination, volitional movement within synergies, or no synergy of shoulder and elbow) were captured through the primary outcomes (FMA-UE motor function) as the evaluation of the hand and wrist could not be performed remotely (requires the physical presence of the assessor). Second, it is important to note the inconsistency in the intervention duration among the participants in the 2 groups (experimental vs control). In the experimental group, 50% (2/4) of the participants received a 3-month intervention and 50% (2/4) received a 2-month intervention. In the control group, 20% (1/5) of the participants received a 3-month intervention, whereas 80% (4/5) received the initial 2-month intervention. That said, it is interesting to note that this variability in duration allowed us to determine the role that the dose (repetition or time spent) played in the recovery. Third, it is important to note that neither the evaluators nor the person in charge of data analysis was blinded to the group assignment. Finally, sex and age factors that may affect exergame use should be further examined using a larger sample size.

In conclusion, the findings of this study should be interpreted with caution, given the small sample size. All explanations provided for the primary and secondary outcomes in both groups remain speculative and need further examination in a larger clinical trial.

### Conclusions

The VirTele intervention constitutes another therapeutic alternative, in addition to the GRASP, to deliver an intense personalized rehabilitation program to survivors of chronic stroke (at least 8 years since the stroke) with UE deficits. Descriptive statistics showed that the highest scores for autonomous motivation were achieved in the experimental group, who achieved a high frequency of use of the exergames and a very high number of repetitions. The study results indicate that the study protocol is valid and can be used to inform larger-scale studies, regardless of the adaptations made because of the context of the COVID-19 pandemic.

## Abbreviations

BCT: behavior change technique
CONSORT: Consolidated Standards of Reporting Trials
FMA-UE: Fugl-Meyer Assessment–Upper Extremity
GRASP: Graded Repetitive Arm Supplementary Program
IREX: Interactive Rehabilitation Exercise
MAL-30: Motor Activity Log-30
MCID: minimal clinically important difference
SDT: self-determination theory
SIS-16: Stroke Impact Scale-16
T1: time point 1
T2: time point 2
T3: time point 3
T4: time point 4
TSRQ-15: Treatment Self-Regulation Questionnaire-15
UE: upper extremity
VR: virtual reality
